# The Impact of Xerostomia on Food Choices—A Review with Clinical Recommendations

**DOI:** 10.3390/jcm12144592

**Published:** 2023-07-10

**Authors:** Frauke Müller, Najla Chebib, Sabrina Maniewicz, Laurence Genton

**Affiliations:** 1 Division of Gerodontology and Removable Prosthodontics, University Clinics of Dental Medicine, 1205 Geneva, Switzerland; 2 Department of Rehabilitation and Geriatrics, Geneva University Hospitals, 1205 Geneva, Switzerland; 3 Clinical Nutrition, Geneva University Hospitals, University of Geneva, 1205 Geneva, Switzerland

**Keywords:** nutrition, nutritional counselling, food choice, xerostomia, hyposalivation, elders, geriatric, gerodontology

## Abstract

Xerostomia and hyposalivation are highly prevalent conditions in old age, particularly among multimorbid elders, and are often attributed to the use of multiple medications. These conditions negatively affect oral functions, such as chewing, swallowing, speech, and taste. Additionally, the lack of lubrication of the oral mucosa frequently leads to super-infections with candida. Denture retention and comfort may also be compromised. The risk of dental caries and erosion of natural teeth increases since saliva, which is essential for repairing initial lesions in tooth structures, is insufficient. The dry sensation in the mouth also impacts the emotional and social well-being of elderly individuals. Patients experiencing xerostomia often avoid certain foods that are uncomfortable or difficult to consume. However, some foods may alleviate the symptoms or even stimulate salivation. This review discusses the limited available evidence on nutritional advice for patients with xerostomia and aims to provide insight into the patient’s perspective while offering clinical recommendations. Future studies should focus on investigating the nutritional intake of individuals suffering from xerostomia or hyposalivation in order to ensure oral health comfort, prevent malnutrition, and minimize the impact on their quality of life.

## 1. Introduction

Xerostomia and hyposalivation are conditions commonly observed in the elderly population, and their occurrence is increasing along with the growing number of elderly individuals in our societies [1]. Xerostomia refers to the subjective sensation of having a dry mouth, while hyposalivation is characterized by a measurable decrease in saliva production [2]. The reported prevalence of xerostomia varies widely, ranging from 5.5 to 78%. These significant differences may be attributable to variations in terminology, survey methods, and specific study populations [1]. In a systematic review with meta-analyses conducted by Pina et al., the prevalence of hyposalivation for unstimulated and stimulated methods was estimated to be 33% (95% CI 21.1–47.0, *p* < 0.0001, n = 2425 individuals) and 30% (95% CI 22.5–39.0, *p* < 0.0001, n = 1495 individuals), respectively [3].

The consequences of reduced saliva production are manifold. They range from difficulties in chewing and swallowing to digestive problems and impaired absorption of nutrients. Moreover, individuals with xerostomia experience a decrease in flavour perception, enjoyment of food, and appetite. Due to the lack of lubrication of the oral mucosa, people with xerostomia often face limitations in their food choices as certain foods become challenging to tolerate, chew, and swallow. The reduced flavour and texture perception can also affect their overall nutritional intake. These consequences may have a significant psychosocial impact and potentially lead to deficiencies in essential nutrients. Saliva plays a vital role in maintaining the health of hard and soft tissues in the oral cavity. It helps remove food debris, neutralize acids, and protect against bacterial infections, thereby preventing dental caries, periodontal disease, and mucositis [3,4]. Hyposalivation promotes the development of dental caries and eventual tooth loss, in particular when combined with poor oral hygiene and a diet rich in sugar and carbohydrates. Therefore, it not only directly affects oral health but also indirectly contributes to impaired masticatory efficiency via the avoidance of various foods with a reduced dentition, which may result in a poor diet and inadequate food intake [5,6,7].

From a patient’s perspective, residents in Long-Term Care facilities who complain at times, or even constantly, of the sensation of a dry mouth state a severe limitation of their oral health-related quality of life [4,8]. Many readers may have personally experienced the unpleasant feeling of a “dry mouth” during stressful situations or while sleeping with an open mouth. The tongue seems to stick to the roof of the mouth, the lips are dry, and speaking is difficult. Eating and swallowing become more challenging, and the sensitive mucosa may limit food choices. Furthermore, even the taste sensation may be affected [9]. The emotional and social impact of xerostomia extends far beyond the oral cavity and undoubtedly has a profound effect on the patient’s daily life and well-being [4].

The aetiology of a dry mouth can be quite diverse. The most severe cases of dry mouth, accompanied by significant hyposalivation, can arise from surgical removal of the salivary glands or radiation therapy as part of cancer treatment [10,11]. Pathologically reduced salivary secretion is also observed in conditions such as Sjogren’s syndrome, diabetes, or Parkinson’s patients [1,12,13]. Further risk factors for varying degrees of salivary deficiency or the sensation of a dry mouth are overall poor health and the use of certain medications. Numerous drugs can impact salivary production, such as antidepressants, anxiolytics, antihypertensives, diuretics, painkillers, medication for Parkinson’s disease, and neuroleptics. Female gender, age, and obesity are other known risk factors in younger patients [14]. Smoking, excessive coffee consumption, heavy snoring, mouth breathing, or dehydration also foster hyposalivation [1,15,16]. Lastly, in elders, the reduced sensation of thirst should also be mentioned, which often leads to insufficient fluid intake and dehydration [17].

Despite ongoing advancements in medicine and dentistry, the available therapeutic options for treating dry mouth are unfortunately still very limited and often unsatisfactory for the patient. Various therapeutic approaches have been described, including saliva stimulation or substitution, medications, or even transcutaneous electrical stimulation, and acupuncture [18,19,20]. Drug intervention to increase salivation seems most adequate for multimorbid elders, but it should be administered by the attending physician rather than within a dental setting. Certain medications, such as pilocarpine or cevimeline, can stimulate the residual capacity of salivary gland tissue. However, improvement of salivary gland hypofunction may be limited [21]. Exciting prospects for future therapeutic options lie in the fields of developmental and stem cell biology [22]. Recently, guidelines for non-nutritional therapies for xerostomia have been published by The Multinational Association of Supportive Care in Cancer/International Society of Oral Oncology, and the American Society of Clinical Oncology [11].

The impact of xerostomia on food choices and dietary patterns is still not well understood. However, providing clinical recommendations can help individuals with xerostomia avoid pain, discomfort, and difficulties with swallowing. Avoiding certain foods may lead to an unbalanced and unhealthy diet, and it may even contribute to malnutrition. On the other hand, making appropriate dietary choices may not only help to alleviate symptoms but may even stimulate salivation. Therefore, the aim of this review is to summarize the impact of xerostomia and hyposalivation on patients’ food preferences and propose clinical recommendations for an adapted diet that is comfortable and safe to chew and swallow, alleviates discomfort, and increases the natural flow of saliva.

## 2. Physiology of Salivation

Saliva primarily consists of water, but it also contains important components essential for maintaining the health of the oral cavity. Alongside electrolytes such as bicarbonate, calcium, fluoride, phosphate, potassium, and sodium, saliva contains various immunoglobulins. Saliva also contains immune factors and phagocytes. Additionally, amylase and growth proteins have been detected in the mucins. The saliva mixture may also contain microorganisms and epithelial cells [23,24]. The functions of saliva extend beyond simply moistening the oral mucosa. Saliva is involved in initiating food digestion, buffering the pH value, remineralising the tooth structure, diluting flavours, and increasing the lubrication of the food to facilitate swallowing. Saliva also has essential functions with regard to the digestive process in the upper parts of the gastrointestinal tract [7].

The major and minor salivary glands of the orofacial system collectively produce approximately 0.5–1.0 L of saliva every day. While the sublingual and submandibular salivary glands produce primarily viscous and highly mucinous saliva, the parotid gland secretes almost 90% of the total saliva of predominantly serous consistency [25]. At rest, without simultaneous chewing, the salivary flow rate is approximately 0.3–0.4 mL per minute. However, during chewing, the salivary flow increases to 1.5–2.0 mL per minute, especially when chewing on one side only [26]. This reflexive secretion of saliva is triggered by the periodontal receptors, which are mechanically stimulated by pressure exerted during chewing on the occlusal surfaces of the teeth [27]. Interestingly, this reflex salivary secretion also occurs in edentulous patients wearing full dentures, and increasing the occlusal force by restorative means can help to increase the salivary flow [28]. The saliva produced during mastication is rather serous and is mostly secreted by the parotid gland. Salivary secretion can also be triggered by gustatory or visual stimuli, such as appetizingly prepared and fragrant food. Thermal or painful stimuli can also stimulate the flow of saliva.

Interestingly, solid objects that are not food may also trigger increased saliva secretion in the oral cavity. This can be observed, for example, after the insertion of dentures [29]. Patients perceive this undesirable side effect of newly fitted dentures as often bothersome, as they may need to swallow more frequently and may have concerns about unnoticed drooling. However, if the stimulus—in this case, the prosthesis—is continuously present, i.e., if the prosthesis is worn consistently for extended periods of time, the reflex adapts, and the salivary flow typically normalizes within the first week after denture insertion. Moreover, replacement dentures can allow for higher chewing forces, which in turn can also increase salivary flow [28]. This effect may persist for a longer duration, although evidence to support this assumption is lacking.

## 3. Age-Related Changes

There is increasing debate regarding the notion that dry mouth is more prevalent in older individuals as a result of physiological age-related changes [30]. Throughout life, the number of glandular cells decreases by approximately 40%, i.e., the ratio of glandular cells to excretory ducts is shifted in favour of the latter [31,32]. Instead of glandular cells, connective tissue and individual fat cells are observed in older people [33]. The stimulation of salivation via the periodontal receptors is also diminished along with lower chewing forces and fewer natural teeth in older adults [34]. However, these changes seem to primarily affect the Stimulated Salivary Flow (SSFR), while resting saliva (RSFR) in healthy elders is equally diminished, but remains adequate to maintain proper oral mucosa moisture and the oral cavity healthy until old age [20]. With advancing age, the number of chronic diseases and the associated intake of medication increase [35], many of which have the undesired effect of causing hyposalivation [36]. Despite a higher prevalence of xerostomia and hyposalivation in old age, it has to be considered a pathological condition rather than a physiological one [37].

## 4. Definitions

Xerostomia refers to the subjective sensation of having a dry mouth, which can occur with or without an actual decrease in salivary flow. On the other hand, hyposalivation refers to an objectively reduced salivary flow. A resting saliva flow rate (RSFR) below 0.1 mL/min or a stimulated saliva flow rate (SSFR) lower than 0.7 mL is commonly regarded as pathological. Monitoring the salivary flow during routine dental check-up visits can be beneficial as a reference to assess impairment [20].

## 5. Clinical Symptoms

The clinical manifestations of hyposalivation comprise dry mucous membranes that may stick to the mirror during a dental examination. The mucosal membranes appear pale, thin, and lack lustre. The tongue often exhibits deep grooves, while the lips feel dry and sticky during speech, a phenomenon often accompanied by an inflammation of the corners of the mouth known as cheilitis angularis. Hyposalivation patients are more prone to candidiasis [38]. If natural teeth are present, they may exhibit a dull surface and fine hairline cracks on the enamel, resembling the appearance of an antique porcelain vase. Erosion, abrasion, and caries are common, with root caries being a particular concern in patients with receding gums, especially when combined with a diet rich in sugar and carbohydrates [39]. The saliva itself appears viscous and is difficult to extract by massaging the parotid gland. Clinically, one may observe whitish, sticky saliva with small bubbles at the corner of the mouth. Difficulties chewing and swallowing specific foods are also common signs of hyposalivation [40].

## 6. Quantifying Salivary Flow

Several methods have been described to test the salivary flow rate during chewing (SSFR) and at rest (RSFR). For the stimulation of saliva flow, Kohler and Winter described a simple test in 1985 by chewing a gauze sponge for 2 min and weighing it before-and-after mastication [41]. Another method proposed by the group of Wolf utilizes the weight loss of candy as a quick measure of hyposalivation [42]. Similarly, chewing gum or paraffin can also be used to stimulate the salivary flow, and the saliva can be collected by spitting into a measuring cup over a given period of time [43]. If specifically targeting the parotid gland, saliva can be collected using a Lashley cup [44]. Unstimulated saliva can be obtained through methods such as draining, spitting, or suction [2,20].

## 7. Patient’s Perception

Patients with xerostomia commonly experience subjective difficulties in speaking due to dry lips and the sensation of the tongue sticking to the roof of the mouth. They also report a decreased sense of taste, leading to a preference for stronger seasonings in meals [45,46]. Chewing and swallowing can be affected, and the oral mucosa may become sensitive or even painful. Flavours are less diluted, making it challenging to tolerate hot spices and acids. Eating dry foods like bread, biscuits, or toast may be difficult. Swallowing becomes a challenge but can be facilitated by taking a sip of liquid along with the food. In denture wearers, the lack of sufficient salivary film can affect denture retention, and inflamed denture-bearing tissues can cause discomfort. In severe cases, denture intolerance may occur.

Validated instruments for assessing xerostomia from the patient’s perspective are important in understanding the impact of dry mouth on their daily lives. As a gold standard, Thomson and co-workers described the “Xerostomia Inventory”, in which 11 problems are rated on a scale from 1 (never) to 5 (very often) [47]. The questions on the Xerostomia Inventory are:

- My mouth feels dry.
- I have problems eating dry foods.
- I get up at night to drink.
- My mouth feels dry during a meal.
- I drink liquids to make swallowing easier.
- I suck on sweets to help with dry mouth.
- Some foods are hard for me to swallow.
- My facial skin feels dry.
- My eyes feel dry.
- My lips feel dry.
- The inside of my nose feels dry.

## 8. Dry Mouth and Nutrition

Reduced salivary flow and the subjective perception of a dry mouth can impair eating and swallowing and lead to oral discomfort, as shown by Dormenval and co-workers in 82 hospitalised geriatric patients [48]. Saliva deficiency can also significantly impact taste perception. The taste buds on the tongue detect taste stimuli and transmit signals to the central nervous system through the chorda tympani, a branch of the seventh facial nerve. When saliva is lacking, the transmission of taste signals may be compromised. Matsuo and co-workers conducted an animal experiment in which the salivary glands were surgically removed from some rats [49]. All four stimuli, salty, sour, bitter, and sweet, that were presented to the experimental animals showed lower activity potentials in the chorda tympani when the salivary glands had been removed.

Measuring the impact of xerostomia and hyposalivation on food choice in a medical setting requires one of the above-mentioned methods to quantify salivary flow. Other approaches comprise the use of questionnaires or monitoring the patient’s nutritional intake over a given period of time. In most cases, no baseline information would be available as a reference; hence, a before-and-after questionnaire may be necessary, despite the shortcomings of retrospective reporting. In addition, psychological instruments and PROMs might evince the impact of the altered diet on the patient’s well-being and quality of life.

Several studies relying on food questionnaires suggest that xerostomia affects the quantity and quality of food intake and ultimately the quality of life [50,51,52]. For instance, in 1405 adults living in Lithuania, xerostomia was associated with lower intakes of carbohydrates and proteins [51]. People aged > 65 years with xerostomia have reduced intakes of omega-3 fatty acids, micronutrients (vitamin E, folate, fluoride), and water [53]. Older studies reported that people with xerostomia tended to avoid crunchy, dry, and sticky foods [52] and had lower intakes of fibre, potassium, vitamin B6, iron, calcium, and zinc [50]. These changes in nutritional intake place people with xerostomia at risk of malnutrition. The European Society of Clinical Nutrition and Metabolism (ESPEN) guideline in geriatrics acknowledges this risk [54,55]. In cases of malnutrition or risk of malnutrition, this guideline provides guidance for nutritional support and recommends strategies such as reducing or replacing medications that contribute to xerostomia. Furthermore, it recognises xerostomia as a risk factor for dehydration. Dehydration can be treated by oral fluid intake in asymptomatic patients with a serum measured plasma osmolality > 300 mOsm/kg but may require subcutaneous or intravenous hydration in cases of more severe dehydration or failure of sufficient oral fluid intakes.

## 9. Nutritional Advise for Relief of Symptoms

While scientific evidence for nutritional recommendations specifically tailored to xerostomia may be limited, there are some general guidelines that can help alleviate symptoms and support overall oral health. In addition to ensuring adequate calorie, protein, and fluid intake, nutritional advice should aim to relieve symptoms. To date, these mostly rely on anecdotal evidence and testimonies of patients on the internet or recommendations on websites of learned societies, associations, or dental practices specialised in hyposalivation treatment, cancer associations, or simply derived from common sense. Hence, the derived recommendations listed in Table 1 and Table 2 are non-exhaustive and not based on scientific evidence (Table 1 and Table 2). Of note, smoking should be discouraged as it worsens xerostomia.

Liquid intake is particularly important for dry mouth patients. Meals should be well-seasoned, although hot and spicy condiments that irritate the mucous membrane should be avoided. As a general rule, meals should be served with lots of gravy. Dry foods, such as biscuits, can be better enjoyed with fruit or green tea. Black tea and coffee, as well as alcohol, on the other hand, increase dehydration and should not be excessively consumed. Thick foods such as yoghurt or ice cream relieve dry mouth symptoms. Sucking lozenges should not be too acidic or contain sugar, as the latter increases the risk of caries and tooth loss. Although acidic fruits may be painful for inflamed mucous membranes, they can still be enjoyed when steamed or baked. High-water-content fruits like watermelon are also recommended. If the mucous membrane is already painfully inflamed, ice cream, an ice cube, or even lozenges from frozen pineapple can cool and soothe. For severe cases, like oncology patients, a viable option is baby food in jars, as it is easy to eat and provides essential nutrients.

Patients need to be advised that mushy and sticky foods should be avoided as they stick to the mucous membranes and the formation of a cohesive bolus may be difficult. When not cleared by the tongue and cheeks, the remaining food can also lead to tooth decay and inflammation of the periodontal tissues, especially if oral hygiene is not performed thoroughly because of the sensitivity and painfulness of the mucous membranes. It should also be avoided to treat the mucous membranes with petroleum-based ointments, such as Vaseline. These dry out the oral mucosa even further and prevent the natural washing away of pathogenic germs [56]. It is important to know that the combination of xerostomia and poor oral hygiene can lead to rapidly progressing root caries and tooth loss within a very short time. Patients with dry mouth should therefore always receive dental care and nutritional advice [57].

The last and probably least satisfactory relief for dry mouth is moistening the oral cavity with small sips or spray shots from a vaporizer of water. Tea, gels, or mouthwashes during the day may provide immediate, but not long-lasting, relief. Ultimately, replacement with artificial saliva is recommended. It is important to consult with a healthcare professional, such as a dentist or doctor, to determine the most suitable artificial saliva product and usage instructions based on individual needs and preferences. They can provide guidance on the appropriate application and frequency of artificial saliva use for optimal relief.

## 10. Nutritional Advise for Stimulating Saliva

Amidst all nutritional guidance, as aforementioned, the utmost important advice is to ensure an adequate intake of fluids, with small “reminders” encouraging the patient to drink a sufficient quantity throughout the day.

Furthermore, some other nutrients may help stimulate salivary flow, such as visco-elastic foods that increase salivation by stimulating the periodontal receptors via occlusal load during chewing (Table 2). Salivation can also be stimulated by unilateral chewing of sugar-free gum [27]. The salivation effect of chewing gum was mentioned already, but regrettably, the act of chewing gum is not particularly favoured by the elderly population. At the age of 85, 6 out of 10 Swiss people wear removable dentures [58]. In this regard, it is unfortunate that all chewing gums presently accessible on the market adhere to denture acrylic, thus rendering the act of chewing exceptionally arduous. This problem may be overcome by recommending silicone tubes of different hardness and surface textures with a large handle to hold. These tubes were conceived to train the chewing muscles during facial growth in children with Duchenne syndrome. However, they may also be used to stimulate salivary flow by unilateral mastication. In this regard, geriatric patients with poor chewing efficiency should not be promptly prescribed a mixed diet, as the benefits of chewing solid food are evident. Before doing so, a dental examination should verify if the patient’s capacity to eat a normal diet can be regained by restorative means.

Salivation can also be stimulated by sucking on sweets, although sour drops may cause dental erosion and are therefore less suitable than aromatic lozenges or liquorice. Care should also be taken to ensure that they contain no sugar to prevent the development of tooth decay.

Recent trends in geriatric medicine confirm a strategy to prioritize medications to reduce the total number of prescribed drugs [59].

## 11. Summary of Recommendations

Saliva production is stimulated by a chewing-active diet. Chewing sugar-free chewing gum in addition to chewing-active main meals is therefore one of the most effective forms of therapy. Meals should be well-seasoned, avoiding excessive spiciness or acidity. Coffee and black tea should only be consumed in moderation. If the mucous membranes are already inflamed, soothing relief can be found in ice cream and frozen pineapple. It is essential to maintain excellent oral hygiene when consuming soft and sticky foods. Overall, it should be emphasised that sufficient fluid intake throughout the day is, in many ways, the most important food for the elderly.

## 12. Future Research

Given the little to no scientific evidence on the food choice of patients suffering from xerostomia or hyposalivation, clinical research, be it qualitative or clinical, is needed to provide evidence-based clinical recommendations, especially concerning a “chewing-active” diet that may help stimulate salivary function. Furthermore, future studies should explore the impact of xerostomia on nutritional status and, if substantiated, develop interventions based on scientific evidence. Last but not least, the patient’s perspective should be addressed in a scientific manner, ensuring their experiences and viewpoints are considered and incorporated into the research findings.

## Figures and Tables

**Table 1:** Drinks and foods having an impact on xerostomia symptoms.

Drinks That May Alleviate Symptoms
Adequate hydration (sips of water, fruit juice or green tea during and outside of meals) Cold fluids
**Foods That May Alleviate Symptoms**
Soft, moist foods, cool or at room temperature High fat liquids (e.g., gravies, cream, milk, sauces, salad dressings) Soups Ice cream, yoghurt, nutritional supplements Frozen pineapple lozenges, frozen grapes Fruits with high water content (e.g., watermelon) Baby food
**Drinks That May Worsen Symptoms**
Caffeine containing drinks (e.g., sodas, black tea and coffee) Alcohol (including alcohol-containing mouthwashes), as they increase the risk of dehydration Acidic beverages, as they may be painful for the mucosal membranes
**Foods That May Worsen Symptoms**
Dry foods (e.g., bread, biscuits, toasts, cake, crackers) Hot, spicy and salty foods Acidic foods (e.g., fresh fruits), as they may be painful for the mucosal membranes Mechanically irritating foods Mushy and sticky foods (e.g., banana, dried fruits, chocolate, honey, jams)

**Table 2:** Foods having stimulating saliva secretion.

**Foods/Chewing Devices That May Foster Salivary Flow**
Sugar-free herbal lozenges or liquorice Moderately acidic and preferably sugar-free candies, beverages and sherbets Non-acidic chewing-active visco-elastic foods (chewing gum or Chewy Tubes^®^)

## Data Availability

Not applicable.
